# The clinical impact of using complex molecular profiling strategies in routine oncology practice

**DOI:** 10.18632/oncotarget.24757

**Published:** 2018-04-17

**Authors:** Jean-François Laes, Philippe Aftimos, Philippe Barthelemy, Joaquim Bellmunt, Guy Berchem, Carlos Camps, Ramón de las Peñas, Ana Finzel, Jesús García-Foncillas, Petteri Hervonen, Ibrahim Wahid, Timo Joensuu, Louis Kathan, Anthony Kong, James Mackay, Christos Mikropoulos, Kefah Mokbel, Jean-Loup Mouysset, Sergey Odarchenko, Timothy J. Perren, Rika Pienaar, Carlos Regonesi, Shadi Salem Alkhayyat, Abdul Rahman El Kinge, Omalkhair Abulkhair, Khaled Morsi Galal, Hady Ghanem, Fadi El Karak, Angel Garcia, Gregori Ghitti, Helen Sadik

**Affiliations:** ^1^ OncoDNA SA, Office: 1, Rue Louis Breguet, Gosselies, Belgium; ^2^ Medical Oncology Clinic, Institut Jules Bordet, Université Libre de Bruxelles, Brussels, Belgium; ^3^ Hôpitaux Universitaires de Strasbourg, Strasbourg, France; ^4^ Institut Hospital del Mar d’Investigacions Médiques, Barcelona, Spain; ^5^ Harvard University, Cambridge, Massachusetts, USA; ^6^ Centre Hospitalier de Luxembourg, Luxembourg, Luxembourg; ^7^ Consorcio Hospital General Universitario de Valencia, Valencia, Spain; ^8^ Consorcio Hospitalario Provincial de Castellón, Castellón, Spain; ^9^ Fundación Jimenez Díaz, IDC Salud, Madrid, Spain; ^10^ Docrates Cancer Center, Helsinki, Finland; ^11^ Pantai Hospital Kuala Lumpur, Kuala Lumpur, Malaysia; ^12^ Cancercare, Cape Town, South Africa; ^13^ University Hospital Birmingham NHS Trust, University of Birmingham, Edgbaston, UK; ^14^ The London Breast Clinic, London, UK; ^15^ Kent Oncology Centre, Kent, UK; ^16^ The Princess Grace Hospital, London, UK; ^17^ Clinique Rambot-Provençale, Aix-en-Provence, France; ^18^ Vinnitsa Regional Clinical Oncology Center, Vinnitsa, Ukraine; ^19^ St. James University Hospital, Leeds, UK; ^20^ Clinica Las Condes, Santiago, Chile; ^21^ King Abdulaziz University Hospital, Jeddah, Saudi Arabia; ^22^ Specialized Medical Center Hospital, Riyadh, Saudi Arabia; ^23^ Kasr El-Aini Hospital, Cairo University, Cairo, Egypt; ^24^ Lebanese American University Medical Center-Rizk Hospital (LAUMC-RH), Beirut, Lebanon; ^25^ Saint Joseph University, Hôtel-Dieu de France University Hospital, Beirut, Lebanon; ^26^ Nuffield Hospital, Chester, UK

**Keywords:** molecular profiling, solid tumour, precision medicine, next-generation sequencing, therapeutic decision making in oncology

## Abstract

Molecular profiling and functional assessment of signalling pathways of advanced solid tumours are becoming increasingly available. However, their clinical utility in guiding patients’ treatment remains unknown. Here, we assessed whether molecular profiling helps physicians in therapeutic decision making by analysing the molecular profiles of 1057 advanced cancer patient samples after failing at least one standard of care treatment using a combination of next-generation sequencing (NGS), immunohistochemistry (IHC) and other specific tests. The resulting information was interpreted and personalized treatments for each patient were suggested. Our data showed that NGS alone provided the oncologist with useful information in 10–50% of cases (depending on cancer type), whereas the addition of IHC/other tests increased extensively the usefulness of the information provided. Using internet surveys, we investigated how therapy recommendations influenced treatment choice of the oncologist. For patients who were still alive after the provision of the molecular information (76.8%), 60.4% of their oncologists followed report recommendations. Most treatment decisions (93.4%) were made based on the combination of NGS and IHC/other tests, and an approved drug- rather than clinical trial enrolment- was the main treatment choice. Most common reasons given by physicians to explain the non-adherence to recommendations were drug availability and cost, which remain barriers to personalised precision medicine. Finally, we observed that 27% of patients treated with the suggested therapies had an overall survival > 12 months. Our study demonstrates that the combination of NGS and IHC/other tests provides the most useful information in aiding treatment decisions by oncologists in routine clinical practice.

## INTRODUCTION

Integration of genomic, transcriptomic and protein analyses is changing the diagnostic landscape of oncology [1–4]. A better choice for chemotherapies, targeted therapies and immunotherapies based on either specific genetic alterations, unusual protein expression or other biomarkers can be more effective and less toxic and costly [5]. This has been successfully demonstrated for a number of therapeutics targeting the protein products of specific genes that are altered in human solid cancers, such as Erb-B2 Receptor Tyrosine Kinase 2 (ERBB2 or HER-2/neu) for trastuzumab, B-Raf Proto-Oncogene, Serine/Threonine Kinase (BRAF) for vemurafenib and dabrafenib, epidermal growth factor receptor (EGFR) for EGFR tyrosine kinase inhibitors or anti-EGFR antibodies, O-6-Methylguanine-DNA Methyltransferase (MGMT) promoter methylation status for temozolomide and, more recently, the expression of programmed death (PD) ligand 1 (PD-L1) for anti-PD1 or anti-PD-L1 therapies in some solid tumours.

In addition to approved therapies, off-label indications and drugs being investigated in clinical trials can be used for treatment if there is knowledge of alterations in genes and protein expression that would drive the development and survival of the tumour [6]. Because the nature and functional effect of mutations and unusual protein expressions are unique to the cancer type and specific to its tumour microenvironment [7], it is critical to provide the most comprehensive overview of all this information in each patient’s cancer. Only then, a personalised treatment plan can be developed, which takes advantage of the clinical evidence regarding all the actionable alterations identified and how tumours harbouring them respond to the growing number of targeted/immune therapies alone or in combination with traditional chemotherapies.

The final goal of providing individualised information is to help oncologists choose the best treatment based on the most relevant information while minimising the amount of irrelevant information they are exposed to. To fulfil this aim, there are several challenges that need to be overcome: (i) each tumour contains inherited (germline) and tumour-specific (somatic) variants. Typically, only a few of the alterations are drivers, and so it is crucial to remove those that are only polymorphisms (not contributing to tumour progression). To eliminate inherited polymorphisms, sequencing of germline DNA is a feasible approach, but only when the focus is on either hotspot mutations or a small panel of genes. However, it is rarely performed routinely for cost reasons. With the increased number of genes included in cancer screening, the identification of those polymorphism (passenger) variants is becoming a bigger challenge. An important point to highlight here is that the necessity of identifying these polymorphisms is not only to determine the real driver mutations, but also for the use of those passenger mutations as surrogates for tumour monitoring through liquid biopsy [8]; (ii) many tests are exclusively focussed on sequencing but the expression of some proteins or the presence of some specific biomarkers could be valuable in defining which strategy to implement and in assisting oncologists in making treatment decisions; (iii) there is uncertainty surrounding whether the receipt of such a complex report influences the oncologist’s treatment decisions, and whether such testing ultimately helps patients.

To evaluate the clinical use of such a multi-dimensional approach, we gathered molecular data of 1057 advanced tumour samples from patients who have already failed at least one standard of care treatment. These samples were analysed by: (i) sequencing of a solid biopsy only, with either a hotspot panel or a comprehensive panel including more than 400 genes; (ii) studying the expression of cancer-related proteins or specific biomarkers, as determined by immunohistochemistry (IHC) and other biomolecular tests (defined as “Package Plus” and described in more detail in the Materials and Methods section); or (iii) the combination of both approaches. In order to determine the utility of tumour profiling in routine clinical practice, we also investigated the final decision made by the oncologists after receiving the report of the molecular characterization of the tumour.

## RESULTS

Using the above approach, we analysed 1057 patients from 30 different countries on four continents, from January 2015 to January 2016. A total of 16,394 different variants were found and classified into the five categories defined in Table 1.

**Table 1:** Definitions of the categories of the variants

Variant category^a^	Definition
Damaging	A variant for which several published studies demonstrated a functional impact on the protein (activating or inhibiting) and where clinical information is also available confirming the impact
Potentially damaging	A variant for which only one publication has shown a functional impact based on an *in vitro* model and for which no clinical information is available
Unknown	A variant for which there are no publications associated with a functional impact and that is not known as a SNP in the NCBI dbSNP database
Polymorphism	A variant identified in the NCBI dbSNP database as a polymorphic variant with a minor allele frequency of at least 1%
Rare polymorphism	A rare polymorphism is a variant found at less than 1% in population but that has been described as benign by functional analysis

^a^Note that this categorization applies comparably to somatic and germline variants.

dbSNP, Single Nucleotide Polymorphism Database; NCBI, National Center for Biotechnology Information; SNP, single nucleotide polymorphism.

**Figure 1:**
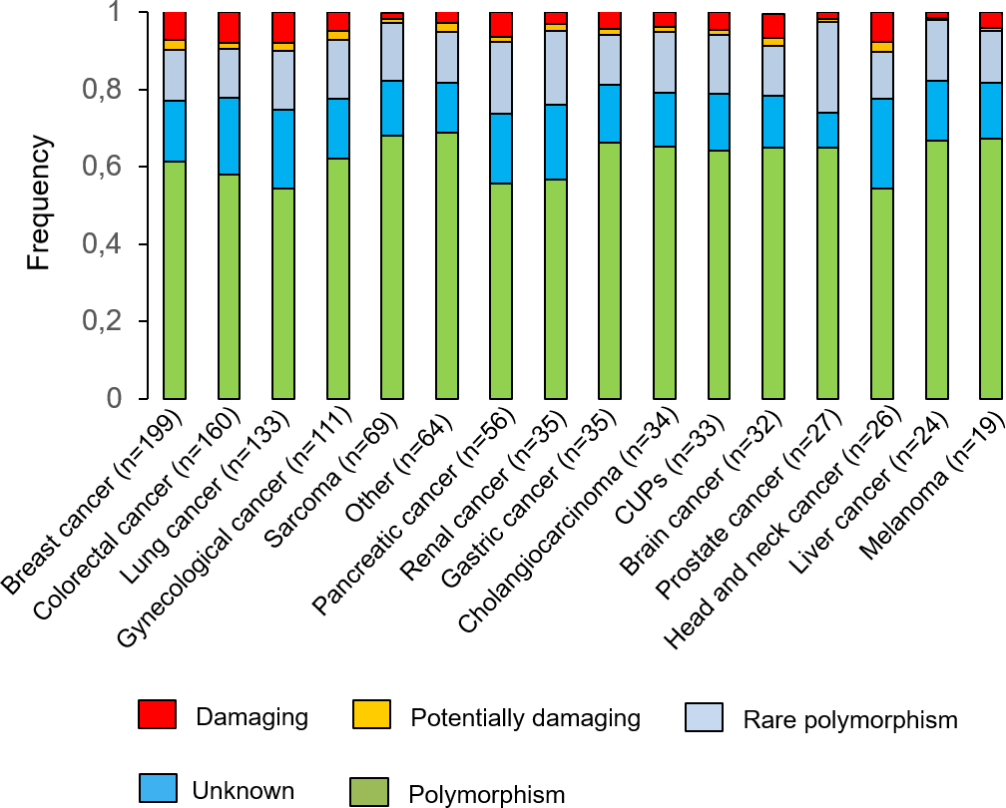
Variants identified by NGS All the variants were stratified by potential functional impact and by cancer type.

After the comparison with the normal diploid population (see Materials and Methods), the percentage of “unknown” variants was reduced by half, independently of the tumour type. Figure 1 shows the resulting different percentages observed for each variant category according to cancer type. Of note, the most mutated genes observed in our analysis were TP53, KRAS and PIK3CA (Supplementary Figure 1).

### The therapeutic impact of the NGS and/or the “Package Plus” tests

We interpreted the data from the NGS and/or the “Package Plus” tests for each patient, and then, based on a review of the clinical information published in the literature, the existing cancer treatments at that period were split into different independent categories based on potential for clinical benefit and on approval status (Table 2). A variant was considered to be associated with a treatment (positively or negatively) if it was damaging and there was clinical evidence for an association reported in the literature. As an example, pembrolizumab was classified as “approved for cancer type” and with “potential clinical benefit” in NSCLC with positive PD-L1 expression.

**Table 2:** Definitions of the treatment categories

Category		Definition
According to approval status		
Approved for cancer type analysed	Treatment approved by the FDA for the tumour type being analysed	
Approved for other cancer type	Treatment approved by the FDA for other tumour but not for cancer type being analysed	
Under development	Treatments in development, tested in phase II, II and IV clinical trials recruiting patients during 2016. These clinical trials (specifying altered genes or pathways within the inclusion criteria) were identified using ClinicalTrials.gov (https://clinicaltrials.gov/).	

According to clinical benefit	
Potential clinical benefit (PCB)	Treatments with evidence for a potential clinical benefit (either associated with a FDA recognized biomarker, present in guidelines or associated with strong clinical evidence). e.g. erlotinib for EGFR-activating mutations in NSCLC, Larotrectinib for NTRK1-fusion solid tumours
Lack of potential clinical benefit (lack of PCB)	Treatments associated with a resistance (e.g. Erlotinib in EGFR T790M mutant NSCLC) or standard of care treatments with no evidence for a potential clinical benefit due to absence of an alteration in the patient (e.g. Pembrolizumab in MSS CRC or in PD-L1 negative NSCLC)
Unknown clinical benefit (unknown PCB)	Treatments with no strong evidence of efficacy (i.e. based only on preclinical data) or with contradictory evidence either found in the literature or based on patient pathway analysis (i.e. contradictory clinical benefit information found for the treatment after comparing the therapeutic impact of alterations detected by next-generation sequencing to those detected in “Package Plus” (IHC) in a sample)
Without treatment	No alternative treatment can be recommended (i.e. no molecular alteration that could predict response or resistance to treatment was detected in the sample)

Categorization according to approval status takes into consideration if the treatment has been FDA approved and in which indications, or if it is still in clinical trial investigation.

Categorization according to clinical benefit evaluates the strength of evidence and the type of response to a treatment based on the presence of a specific molecular alteration.

Note that these 2 categories are independent. A treatment being classified according to the approval status does not automatically qualify it to any of the categories in clinical benefit. Indeed, a FDA approved drug can be classified as “unknown” clinical benefit if there is no strong evidence of clinical benefit to the aberration detected in the sample, or if there is contradictory evidence based on patient pathway analysis.

MSS: microsatellite stable.

**Figure 2:**
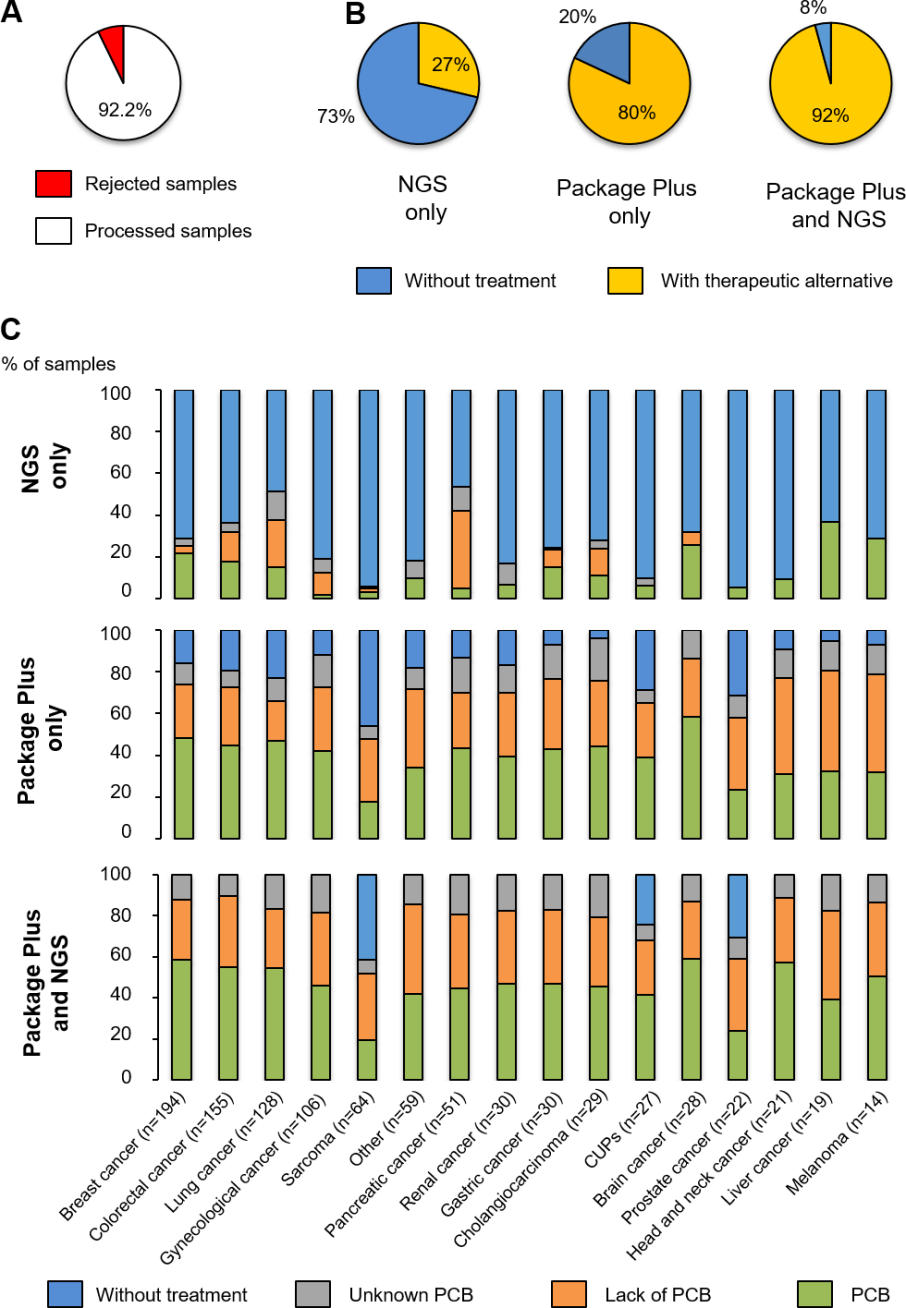
Categorisation of samples analysed (**A**) Number of samples rejected and processed; (**B**) Patients with treatment options (approved for the cancer type analysed, approved for other cancer types or under development) and without, according to test; (**C**) The potential or lack of potential or the unknown potential clinical benefit of samples, according to test and cancer type. PCB, potential clinical benefit.

We observed an average of 7.78% of samples rejected because of not enough quantity or bad quality of the material received (Figure 2A). When the proportion of variants with an associated treatment was determined (Figure 2B), approximately 30% of the samples examined using NGS only were associated with a treatment, but this percentage increased when variants were investigated using the “Package Plus” (80%) and the “Package Plus” and NGS combined (92%). The percentage of useful variants based on NGS only was highly associated with the cancer type, and more specifically with the most common cancers such as breast, colorectal and lung cancers (Figure 2C). In contrast to the data obtained with exclusively NGS, which provided no information on useful treatments in 50% to 90% of the cases, the percentage of useful treatments based on “Package Plus” was greater across the different cancer types (Figure 2C).

When NGS and “Package Plus” were combined, only sarcoma, carcinoma of unknown primary site and prostate cancer had samples that were without any associated treatment (40.7%, 19% and 26.3%, respectively; Figure 2C). All other cancer types were associated with treatment options after combining NGS and “Package Plus” results. Of note, most of the recommended treatments from the combined analysis were targeted therapies (53.8%), followed by chemotherapies (41.4%) and immunotherapies (4.8%). Nivolumab and pembrolizumab were the most recommended immunotherapies; within the category of chemotherapy, the main recommended treatments were taxanes (26%), doxorubicin/epirubicin (23%) and platinum-based therapies (10%); while PIK3CA/mTOR inhibitors (30%), MEK pathway inhibitors (30%) and anti-androgen (3%) were the main suggested treatments in the group of targeted therapy.

### Influence of the molecular profiling results on oncologist´s treatment choice

Survey forms were sent to oncologists 3 months after the molecular profiling results were available, of which 255 were completed, meaning that approximately 25% of the physicians participated. The oncologists who did not complete the survey form cited either an ethical reason (i.e. in their countries, providing an external organisation with such information is not allowed) or a “time” reason (too busy to complete a survey). Answers were received from oncologists worldwide (> 10 countries in 4 continents).

**Figure 3:**
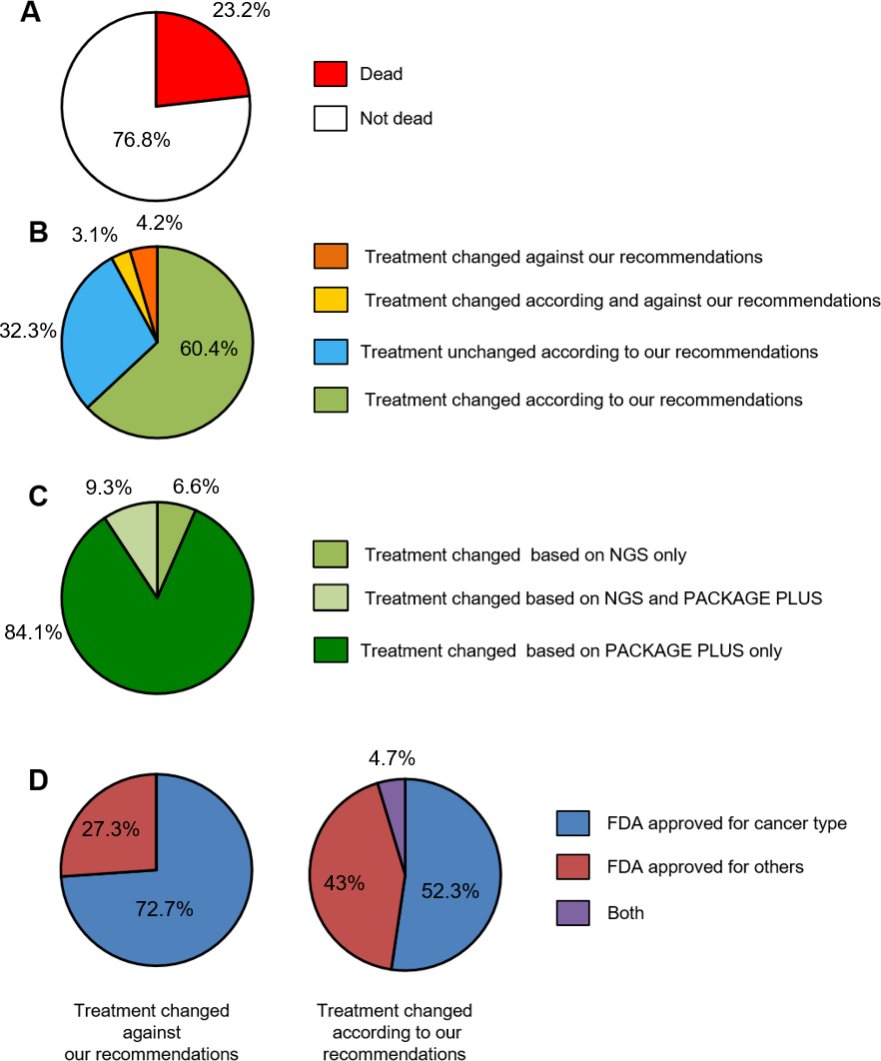
Results of oncologist survey (**A**) Survival status of patients 3 months after provision of molecular results; (**B**) Treatment choices according and against recommendations; (**C**) Treatment modification stratified based on the test type; (**D**) Treatment choices according to treatment approval status.

As shown in Figure 3A, 23.2% of the patients passed away before receiving any new treatment. Of the 76.8% of patients who were alive at the time of the survey, 60.4% of their oncologists followed the report recommendations, 32.3% did not, 3.1% followed some and went against others, and 4.2% decided to treat their patient against report recommendations (meaning that they treated their patient with a treatment considered in the analysis as having a lack of clinical benefit) (Figure 3B).

In order to understand the reason behind the choice of not following the report’s recommendations, the oncologists’ answers were considered in more detail. The three main reasons given were: (i) the treatments advised are not available in my country, (ii) the treatments advised are not affordable in my country or (iii) I already chose a treatment before receiving your report. In this last case, even if our report agreed with the oncologist’s choice, it did not influence their decision.

In those cases for which oncologists provided treatment against recommendations, the answers were as follows: (i) I have other evidence, (ii) I do not have any other choice of treatment available in my country.

Among the cases for which oncologists followed the report recommendations, the vast majority of the decisions were based either on the “Package Plus” only or on the combination of NGS and “Package Plus” (93.4%) (Figure 3C). Only 6.6% of the decisions were based on the NGS results exclusively. We also analysed which treatments were chosen by the oncologists according to approval status (Table 2). Figure 3D shows that all the treatments prescribed by the oncologists were either approved for the cancer type analysed or approved for another cancer type. No investigational drug was chosen, and no one got enrolled in a clinical trial.

**Figure 4:**
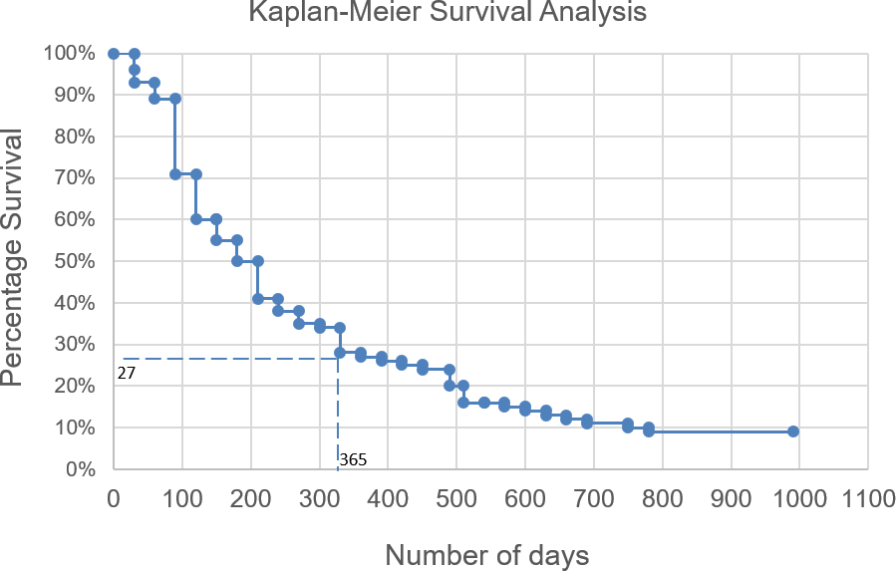
Overall survival of patients For the cases where our recommendations were followed (*n =* 114), the overall survival was analysed.

The follow-up questionnaires were also used to monitor how the information in the report could have impacted overall survival. We observed that in the cases for which our recommendations were followed, at least 50% of the patients had an OS of > 6 months, and 27% of patients had a minimum OS of > 12 months (Figure 4). A lack of treatment response appeared to be mostly due to patients being considered for palliative treatment, where patients at this stage have an average OS of ∼3–6 months [9, 10]; However, this result needs to be confirmed with larger follow-up data and other clinical efficacy endpoints.

## DISCUSSION

Overall, these data provide a comprehensive analysis of a potential precision medicine strategy, and information on its utility compared with objective decisions made in routine clinical practice by the treating oncologists. The results obtained show that the use of matched tumour and normal DNA for genomic analyses is a direct approach to potentially filter out non-pathogenic mutations and hence reduce the amount of irrelevant aberrations provided to physicians by at least 50%. This facilitates the identification of clinically actionable somatic alterations in cancer specimens. However, in daily clinical practice and for cost reasons, only DNA from the tumour is sequenced.

Moreover, these data highlight the added value of using a combination of molecular tests, with the integration of NGS and IHC/other specific tests data providing the most useful information regarding potential treatment options compared with any of those methods alone. The more information derived from the tests performed, the more useful the results can be; for example, in the current study the proportion of variants in prostate cancer for which there was no associated treatment was significantly reduced by the recent addition of the ARV7 test and the sequencing of BRCA1&2 into the “Package Plus” analysis (data not shown). On the other hand, the continued existence of variants with no treatment options in sarcoma and carcinoma of unknown primary in the present study can be explained by the fact that there is no treatment available. However, further larger studies with more detailed survival/treatment response metrics are needed to unravel whether it is the NGS data, “Package Plus” data or the integration of both that could better predict clinical benefit and more effectively impact clinical response.

The results of this work are in accordance with other studies that have also used molecular screening to identify potential personalised treatment options for patients with different advanced solid tumours [11–13]. While there is evidence that the use of precision medicine can improve treatment outcomes [9, 14, 15], barriers to its full implementation in routine clinical practice remain. These include the complexity of the molecular information generated by these tests, uncertainty surrounding the clinical utility of the information, lack of knowledge about precision medicine in general among healthcare professionals, and economic considerations such as cost and reimbursement structures affecting access to these tools [16–20], although the cost of DNA sequencing is decreasing. Moreover, the number of genes that can be sequenced is very large, and not all of these genes will have a practical application; as shown in the current study, the number of variants identified in the tumour samples as potentially useful was reduced by half when the tumour variants were compared with the ones found in a normal population. In the case of precision medicine, more information is not necessarily better, and techniques to reduce the information noise generated by these methods are paramount for increasing the usefulness of molecular information for physicians.

When investigating in this study the usefulness of the information provided to oncologists on their clinical decision-making process, it was observed that approximately two-thirds of treating oncologists followed the recommendations provided in the report. A minority of oncologists surveyed (4.4%) went against the recommendations. In 99% of the cases where our recommendations were not followed (excluding the cases where the oncologists went against them), the reason given was not related to scientific or clinical considerations, but rather to the barriers of drug availability and cost (the treatments are not available in my country; patients cannot afford the treatments because they are not reimbursed in my country) or for practical reasons, such as a lack of clinical trials for the recommended treatment being investigated in the oncologist’s country. The results obtained from the survey regarding treatment options chosen also reflected what is known about drug-related barriers to precision medicine, since in all the cases, the treatment given was either approved for the cancer type analysed or approved for another cancer type (mainly chemotherapies and few targeted or immunotherapies), and unfortunately, despite recommendations, no patient was assigned to clinical trials.

This brings up an important point: many challenges of precision medicine in oncology, i.e. how to choose and deliver the right treatment(s) to the right person at the right time, can be best addressed through well-designed clinical trials. Genomic-based clinical trials (such as umbrella, basket, and adaptive trial design) can maximize the opportunity to allocate patients to the best treatment option based on the molecular profile and to study deeply clinical validity and utility of these allocations [21, 22]. That’s why it was disappointing to find in our study that none of the patients was enrolled in a trial. Besides lack of relevant clinical trials in the patient’s country, other barriers to enrolment that could have driven this decision were: preference of the oncologist to prescribe an approved drug over clinical trial (many of oncologists ordering our tests are from community hospitals and centers, and so this tendency is in accordance with published data [20, 23]); preference to enroll patients only in phase 3 studies (which were not available for all patients); and the very advanced stage of most patients (who had poor ECOG performance after failing multiple treatments) which would disqualify them from most trials.

Interestingly, very few oncologists indicated that they made treatment decisions based on NGS alone, since NGS does not provide a lot of information on chemotherapies, which are the most readily available and cost-effective options available in most countries; this result reinforces the need for a multi-faceted approach towards precision medicine in routine clinical practice.

Limitations of this analysis include its retrospective design and the lack of information regarding what would have been the treatment’s choice of the oncologists based only on their own knowledge and in the absence of the information provided. Another limitation is the low response rate for the follow-up surveys, that has also restricted the collection of important endpoints such as progression-free survival and time on therapy- important metrics to reinforce the value of the molecular tests on clinical utility. On the other hand, strengths include the number of samples analysed and the links made between the information provided and its influence on treatment choice. Further research is required, including the screening of more cancer types and prospective studies into the use and the clinical utility of this type of information to inform subsequent treatment; the questions of how to best treat patients with currently available treatments, how to encourage clinical trial participation, and how to address the issue of drug cost and reimbursement are yet unanswered.

In conclusion, this study suggests that a combination of tests analysing DNA, RNA and protein changes in tumours could represent the best approach for obtaining molecular information that would be useful to oncologists in routine clinical practice for guiding them towards alternative treatments. Despite the provision of this information to oncologists, barriers to full implementation of this approach remain, and include drug availability and cost, and low participation in clinical trials.

## MATERIALS AND METHODS

### Patient population

This work is a retrospective study evaluating 1057 patients from 30 different countries on four continents with an advanced solid cancer who (1) had failed at least one line of therapy for their advanced disease before undergoing molecular profiling; and (2) had their tissue sample tested using OncoDEEP™ (80.43%) or OncoDEEP Clinical™ (19.57%) (OncoDNA, Gosselies, Belgium) profiling solutions from January 2015 to January 2016. All patients were suggested these solutions by their medical oncologists and were consented before the tissue was sent for molecular testing. These patients could have stage III or IV disease, and samples sent for molecular profiling were biopsies taken after progression on therapy and could be the primary (23.1%) or the metastatic (75.3%) tissue (in 1.6% of cases, the origin of the tissue was not specified). For objectivity, all samples were included in our analysis without a prior selection on age, cancer type, prior treatment, profiling results or follow-up data.

### Samples

Tissue samples from different tumour types, as well as blood samples from 200 healthy individuals, were included in the analysis. A large range of cancer types were studied, including breast (18.8%), colorectal (15.1%), lung (12.6%), gynaecological (10.5%), sarcomas (6.5%), pancreatic (5.3%), renal (3.3%), gastric (3.3%), cholangiocarcinoma (3.2%), cancer of unknown primary (3%), brain (3%), prostate (2.6%), head and neck (2.5%), liver cancer (2.3%), melanoma (1.8%) and other rare cancers (6.1%) (Supplementary Table 1). The tumour biopsy sample was formalin-fixed paraffin-embedded (FFPE) tissues that was no older than 6 months. FFPE tissues underwent pathological review to determine if criteria were met: tumour tissue must be > 10% of the whole sample, the size of the tumour must be > 5 mm^2^, and the lymphocytes invasion must be < 20% in the region where the tumour cells are located. Tumours were macrodissected to remove any contaminating normal tissue.

### Sample preparation and next-generation sequencing

DNA was extracted from FFPE tissue or blood using the Qiagen DNA FFPE Tissue Kit or Qiagen DNA Blood Mini Kit (Qiagen, Valencia, CA, USA), respectively. DNA quantity was measured using the Qubit 2.0 Fluorometer (Thermo Fisher Scientific, Waltham, USA).

To identify somatic alterations in tumour samples, we designed two Ampliseq custom panels (OncoDNA, Gosselies, Belgium) to amplify by NGS, either 207 amplicons covering hotspot mutations of 65 genes (OncoDEEP™: updated version of the Ion Ampliseq Cancer Hotspot Panel v2) or > 16,452 amplicons covering whole exons of 409 genes (OncoDEEP Clinical™: Ion Ampliseq Comprehensive Cancer Panel) (Supplementary Table 2). Briefly, the targeted sequencing libraries were generated using the Ion AmpliSeq Library kit 2.0 according to the manufacturer’s instructions (Thermo Fisher Scientific, Waltham, USA). The starting material consisted of either 10 ng (OncoDEEP™) or 50 ng (OncoDEEP Clinical™) from FFPE samples depending on the panel chosen, while normal DNA extracted from blood was always analysed with OncoDEEP Clinical™. The primers used for amplification were partially digested by the Pfu enzyme. The product of digestion was then ligated with corresponding barcoded adapters and purified using Ampure Beads (Agilent Genomics Inc). The product of purification was amplified for 5 more cycles and subsequently purified using Ampure Beads. The quality of the libraries was assessed using the Qubit dsDNA HS Assay kit (Thermo Fisher Scientific, Waltham, USA). 10 pM of each library was loaded into the IonChef system (Thermo Fisher Scientific, Waltham, USA) for the emulsion polymerase chain reaction (PCR) and then loaded in the chip. An average coverage of 1000x was targeted to be able to detect variants down to 5% for the FFPE and blood samples. We used either the *Personal* Genome Machine (PGM), the Proton or the 5XL devices (Thermo Fisher Scientific, Waltham, USA) to sequence depending on the required throughput.

### Primary processing of next-generation sequencing data and identification of putative somatic mutations

The data generated from the FFPE and normal DNA samples were first aligned to the human reference sequence and annotated using the Consensus Coding DNA Sequences (CCDS), RefSeq, and Ensembl databases. NGS data were then analysed by using the Torrent Suite Software (Thermo Fisher Scientific, Waltham, USA). Next, somatic mutations were identified with the Variant Caller 4.0 software using the somatic high stringency parameters (Thermo Fisher Scientific, Waltham, USA) to ensure sufficient coverage of the analysed bases and to exclude mapping and sequencing errors (Supplementary Table 3). Mutation analysis was focussed on single-base substitutions as well as small insertions and deletions. Candidate somatic mutations were further filtered based on: coverage of > 100; a forward-reverse ratio of 10%, 90%; gene annotation to identify those occurring in protein-coding regions; the exclusion of intronic and silent changes; and the retention of mutations resulting in missense mutations, nonsense mutations, frame shifts, or splice site alterations. A manual visual inspection step was used to further remove artefactual changes. Mutations were separated into those associated with a described biological impact on the function of the proteins and those common germline mutations found in the NCBI dbSNP human variation sets in VCF (variant call format) version 138 (https://www.ncbi.nlm.nih.gov/variation/docs/human_variation_vcf/) labelled as “common” with a germline minor allele frequency of ≥ 0.01 and annotated as having “no known medical impact.” The remaining mutations (with no described biological impact and not present in NCBI dbSNP database) were compared to the set of variants identified in the normal diploid population (blood samples) and labelled as “polymorphism” if found in it.

### Analysis of immunohistochemistry results and other biomolecular tests

For 80% of the patients, an additional bundle of IHC (Supplementary Table 4) and other molecular tests were performed (called “Package Plus”). All the alterations tested in this package are directly related to patient´s response towards different kinds of treatment. Moreover, this tests’ combination is personalized to the patient since it takes into account the tumour type, the biomarkers already tested and the previous treatment[s] given. Each IHC was analysed by microscopy in a double-blind fashion. A score was calculated based on a predefined ISO-accredited scoring method (which is IHC dependent) and the consensus of both analyses was used to define the level of expression or activation of the proteins. The other biomolecular tests considered were methylation of the MGMT promoter, the expression of either EGFRVIII, MET Proto-Oncogene, Receptor Tyrosine Kinase (MET)-exon 14 deletion or androgen-receptor splice variant 7 messenger RNA (ARV7) as determined by quantitative PCR, and microsatellite instability testing by Genescan^®^ analysis (Thermo Fisher Scientific, Waltham, USA).

### Clinical relevance of the analyses

A literature search was performed to identify FDA (Food and Drug Administration) labels, official guidelines (National Comprehensive Cancer Network), and retrospective and prospective clinical studies (published in PubMed or as conference abstracts), pertaining to genomic alterations of each gene and their association with outcomes in cancer patients, and to remove variants not known to be damaging or potentially damaging (Table 1). To keep our internal variant database updated with the newest published data on these mutations, an automatic search was done daily, and then its results were curated by scientific experts. Variants were sorted into four categories according to their impact on the functionality of the corresponding protein (Table 1). This functional impact classification of the mutations and their clinical actionability was then compared to that of OncoKB [24]. OncoKB is an openly accessible, expert-guided precision oncology knowledge database that assigns each variant to 1 of 5 levels of evidence corresponding to its actionability. For this analysis, variants were considered actionable and associated with clinical benefit if they ranked as levels 1–3 (i.e. associated with a standard therapy or investigational therapy) or level R1 (i.e. associated with resistance to a standard therapy) in OncoKB.

Moreover, all tests included in the “Package Plus” investigate alterations that rank as levels 1–3 or R1. The treatments recommended for each patient according to the molecular profile fell into three different categories: “Approved for cancer type analysed”, “Approved for other cancer type” and “Under development” (Table 2).

Of note, once the patient’s sample passed the quality control (explained above), it takes a maximum of seven working days for the report (that includes treatment/clinical trial suggestions) to be ready. The physician can access the report via a proprietary online interface, and download it as a PDF document.

### Oncologist survey

To determine whether the provision of the tumour genetic profiling, immunohistochemistry and other biomolecular test results influenced oncologist´s treatment decisions, an automatic system (web-based) was developed to ask the following questions:

1. Did the patient pass away before the application of the treatment?
2. Have you changed the treatment decision on the basis of the results generated by OncoDEEP™/OncoDEEP Clinical™?
3. Have you prescribed one or several drugs suggested in our report (either approved or tested in clinical trial)? If yes, which one(s)? If not, why?

These questions were sent to the oncologists via email 3 months after the biomolecular results were available. Participation in the survey was voluntary and uncompensated.

New surveys were automatically sent after the initial one every 3–4 months to follow-up on how the patient was responding to treatment. There were 114 cases where the oncologist who followed our therapy recommendations provided us with the follow-up information, either until cut-off of the analysis (January 2016) or until patient deceased. For these 114 cases, overall survival (OS) was analysed. OS was defined as the time from initial testing (study enrolment) to January 2016 or to death due to any cause.

## SUPPLEMENTARY MATERIALS FIGURE AND TABLES





## Abbreviations

NGS: next-generation sequencing
IHC: immunohistochemistry
FFPE: formalin-fixed paraffin-embedded
FDA: Food and Drug Administration
OS: overall survival
PCR: polymerase chain reaction
dbSNP: Single Nucleotide Polymorphism Database
SNP: single nucleotide polymorphism
PCB: potential clinical benefit

## References

[1] Van Allen EM, Wagle N, Stojanov P, Perrin DL, Cibulskis K, Marlow S, Jane-Valbuena J, Friedrich DC, Kryukov G, Carter SL, McKenna A, Sivachenko A, Rosenberg M (2014). Whole-exome sequencing and clinical interpretation of formalin-fixed, paraffin-embedded tumor samples to guide precision cancer medicine. Nat Med.

[2] Frampton GM, Fichtenholtz A, Otto GA, Wang K, Downing SR, He J, Schnall-Levin M, White J, Sanford EM, An P, Sun J, Juhn F, Brennan K (2013). Development and validation of a clinical cancer genomic profiling test based on massively parallel DNA sequencing. Nat Biotechnol.

[3] Garraway LA, Janne PA (2012). Circumventing cancer drug resistance in the era of personalized medicine. Cancer Discov.

[4] Wagle N, Berger MF, Davis MJ, Blumenstiel B, Defelice M, Pochanard P, Ducar M, Van Hummelen P, Macconaill LE, Hahn WC, Meyerson M, Gabriel SB, Garraway LA (2012). High-throughput detection of actionable genomic alterations in clinical tumor samples by targeted, massively parallel sequencing. Cancer Discov.

[5] Stegmeier F, Warmuth M, Sellers WR, Dorsch M (2010). Targeted cancer therapies in the twenty-first century: lessons from imatinib. Clin Pharmacol Ther.

[6] Saiyed MM, Ong PS, Chew L (2017). Off-label drug use in oncology: a systematic review of literature. J Clin Pharm Ther.

[7] Wang M, Zhao J, Zhang L, Wei F, Lian Y, Wu Y, Gong Z, Zhang S, Zhou J, Cao K, Li X, Xiong W, Li G (2017). Role of tumor microenvironment in tumorigenesis. J Cancer.

[8] Siravegna G, Mussolin B, Buscarino M, Corti G, Cassingena A, Crisafulli G, Ponzetti A, Cremolini C, Amatu A, Lauricella C, Lamba S, Hobor S, Avallone A (2015). Clonal evolution and resistance to EGFR blockade in the blood of colorectal cancer patients. Nat Med.

[9] Reljic T, Kumar A, Klocksieben FA, Djulbegovic B (2017). Treatment targeted at underlying disease versus palliative care in terminally ill patients: a systematic review. BMJ Open.

[10] White N, Reid F, Harris A, Harries P, Stone P (2016). A Systematic Review of Predictions of Survival in Palliative Care: How Accurate Are Clinicians and Who Are the Experts?. PLoS One.

[11] Zehir A, Benayed R, Shah RH, Syed A, Middha S, Kim HR, Srinivasan P, Gao J, Chakravarty D, Devlin SM, Hellmann MD, Barron DA, Schram AM (2017). Mutational landscape of metastatic cancer revealed from prospective clinical sequencing of 10,000 patients. Nat Med.

[12] Kato Y, Nishihara H, Yuzawa S, Mohri H, Kanno H, Hatanaka Y, Kimura T, Tanino M, Tanaka S (2013). Immunohistochemical molecular gene expression profile of metastatic brain tumor as a potent personalized medicine. Brain Tumor Pathol.

[13] Nie X, Cheng G, Ai B, Zhang S (2013). The tailored chemotherapy based on RRM1 immunohistochemical expression in patients with advanced non-small cell lung cancer. Cancer Biomark.

[14] Stockley TL, Oza AM, Berman HK, Leighl NB, Knox JJ, Shepherd FA, Chen EX, Krzyzanowska MK, Dhani N, Joshua AM, Tsao MS, Serra S, Clarke B (2016). Molecular profiling of advanced solid tumors and patient outcomes with genotype-matched clinical trials: the Princess Margaret IMPACT/COMPACT trial. Genome Med.

[15] Massard C, Michiels S, Ferté C, Le Deley MC, Lacroix L, Hollebecque A, Verlingue L, Ileana E, Rosellini S, Ammari S, Ngo-Camus M, Bahleda R, Gazzah A (2017). High-Throughput Genomics and Clinical Outcome in Hard-to-Treat Advanced Cancers: Results of the MOSCATO 01 Trial. Cancer Discov.

[16] Vasan N, Yelensky R, Wang K, Moulder S, Dzimitrowicz H, Avritscher R, Wang B, Wu Y, Cronin MT, Palmer G, Symmans WF, Miller VA, Stephens P (2014). A targeted next-generation sequencing assay detects a high frequency of therapeutically targetable alterations in primary and metastatic breast cancers: implications for clinical practice. Oncologist.

[17] Horgan D, Jansen M, Leyens L, Lal JA, Sudbrak R, Hackenitz E, Bußhoff U, Ballensiefen W, Brand A (2014). An index of barriers for the implementation of personalised medicine and pharmacogenomics in Europe. Public Health Genomics.

[18] Najafzadeh M, Davis JC, Joshi P, Marra C (2013). Barriers for integrating personalized medicine into clinical practice: a qualitative analysis. Am J Med Genet A.

[19] Petersen KE, Prows CA, Martin LJ, Maglo KN (2014). Personalized medicine, availability, and group disparity: an inquiry into how physicians perceive and rate the elements and barriers of personalized medicine. Public Health Genomics.

[20] Meric-Bernstam F, Brusco L, Shaw K, Horombe C, Kopetz S, Davies MA, Routbort M, Piha-Paul SA, Janku F, Ueno N, Hong D, De Groot J, Ravi V (2015). Feasibility of Large-Scale Genomic Testing to Facilitate Enrollment Onto Genomically Matched Clinical Trials. J Clin Oncol.

[21] Horak P, Fröhling S, Glimm H (2016). Integrating next-generation sequencing into clinical oncology: strategies, promises and pitfalls. ESMO Open.

[22] Siu LL, Conley BA, Boerner S, LoRusso PM (2015). Next-Generation Sequencing to Guide Clinical Trials. Clin Cancer Res.

[23] Statz CM, Patterson SE, Mockus SM (2017). Barriers preventing the adoption of comprehensive cancer genomic profiling in the clinic. Expert Rev Mol Diagn.

[24] Chakravarty D, Gao J, Phillips SM, Kundra R, Zhang H, Wang J, Rudolph JE, Yaeger R, Soumerai T, Nissan MH, Chang MT, Chandarlapaty S, Traina TA (2017). OncoKB: A Precision Oncology Knowledge Base. JCO Precis Oncol.

