# Supplementary material for: The clinical impact of using complex molecular profiling strategies in routine oncology practice
Source: Oncotarget. 2018 Apr 17;9(29):20282–93. doi: 10.18632/oncotarget.24757 (PMC5945513; doi:10.18632/oncotarget.24757)
Supplement: Supplementary file 1 [file oncotarget-09-20282-s001.pdf]

The clinical impact of using complex molecular profiling strategies in routine oncology practice

SUPPLEMENTARY MATERIALS

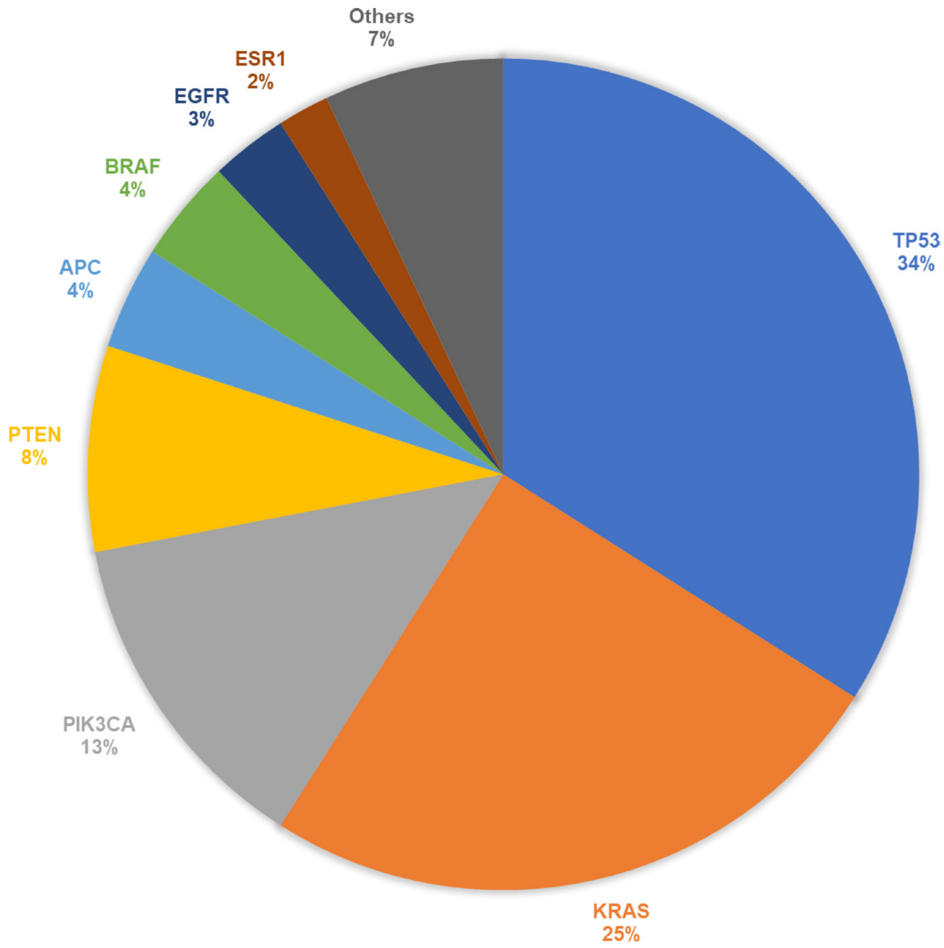

Supplementary Figure 1: Distribution of the most mutated genes observed in this analysis.

Supplementary Table 1: Distribution of the analysed cancer types

Cancer Type		Percentage
Breast cancer		18.8
Colorectal cancer		15.1
Lung cancer		12.6
Gynaecological cancer		10.5
Sarcoma		6.5
Pancreatic cancer		5.3
Renal cancer		3.3
Gastric cancer		3.3
Cholangiosarcoma		3.2
Cancer of unknown primary		3
Brain cancer		3
Prostate cancer		2.6
Head and neck cancer		2.5
Liver cancer		2.3
Melanoma		1.8
Other cancers		6.1
	Adenoid cystic carcinoma	0.57
	Adnexal Skin carcinoma	0.19
	Desmoid tumor	0.1
	Duodenal adenocarcinoma	0.19
	Esophageal cancer	0.95
	Neuroendocrine Tumors	0.57
	Paraganglioma	0.1
	Parathyroid cancer	0.1
	Penile cancer	0.19
	Peritoneal mesothelioma	0.48
	Testicular cancer	0.29
	Thymoma and Thymic carcinoma	0.66
	Thyroid cancer	0.19
	Urinary bladder cancer	1.52

Supplementary Table 2: Genes analysed by next-generation sequencing

Hotspot Genes	ABL1, AKT1, ALK, APC, ATM, AURKA, BRAF, CCND1, CCNE1, CDH1, CDK4, CDKN2A, CSF1R, CTNNB1, DDR2, DPYD, EGFR, ERBB2, ERBB4, ESR1, EZH2, FBXW7, FGFR1, FGFR2, FGFR3, FLT3, GNA11, GNAQ, GNAS, HNF1A, HRAS, IDH1, IDH2, JAK2, JAK3, KDR, KIT, KRAS, MAP2K1, MCL1, MDM2, MET, MLH1, MPL, MYC, NF1, NOTCH1, NPM1, NRAS, PDGFRA, PDGFRB, PIK3CA, PTEN, PTPN11, RB1, RET, SMAD4, SMARCB1, SMO, SRC, STK11, TP53, TPMT, UGT1A1, VHL
Comprehensive Panel Genes	ABL1, ABL2, ACVR2A, ADAMTS20, AFF1, AFF3, AKAP9, AKT1, AKT2, AKT3, ALK, AMER1, APC, AR, ARID1A, ARID2, ARNT, ASXL1, ATF1, ATM, ATR, ATRX, AURKA, AURKB, AURKC, AXL, BAI3, BAP1, BCL10, BCL11A, BCL11B, BCL2, BCL2L1, BCL2L2, BCL3, BCL6, BCL9, BCR, BIRC2, BIRC3, BIRC5, BLM, BLNK, BMPR1A, BRAF, BRD3, BRIP1, BTK, BUB1B, CARD11, CASC5, CBL, CCND1, CCND2, CCNE1, CD79A, CD79B, CDC73, CDH1, CDH11, CDH2, CDH20, CDH5, CDK12, CDK4, CDK6, CDK8, CDKN2A, CDKN2B, CDKN2C, CEBPA, CHEK1, CHEK2, CIC, CKS1B, CMPK1, COL1A1, CRBN, CREB1, CREBBP, CRKL, CRTC1, CSF1R, CSMD3, CTNNA1, CTNNB1, CYLD, CYP2C19, CYP2D6, DAXX, DCC, DDB2, DDIT3, DDR2, DEK, DICER1, DNMT3A, DPYD, DST, EGFR, EML4, EP300, EP400, EPHA3, EPHA7, EPHB1, EPHB4, EPHB6, ERBB2, ERBB3, ERBB4, ERCC1, ERCC2, ERCC3, ERCC4, ERCC5, ERG, ESR1, ETS1, ETV1, ETV4, EXT1, EXT2, EZH2, FANCA, FANCC, FANCD2, FANCF, FANCG, FAS, FBXW7, FGFR1, FGFR2, FGFR3, FGFR4, FH, FLCN, FLI1, FLT1, FLT3, FLT4, FN1, FOXL2, FOXO1, FOXO3, FOXP1, FOXP4, FZR1, G6PD, GATA1, GATA2, GATA3, GDNF, GNA11, GNAQ, GNAS, GPR124, GRM8, GUCY1A2, HCAR1, HIF1A, HLF, HNF1A, HOOK3, HRAS, HSP90AA1, HSP90AB1, ICK, IDH1, IDH2, IGF1R, IGF2, IGF2R, IKBKB, IKBKE, IKZF1, IL2, IL21R, IL6ST, IL7R, ING4, IRF4, IRS2, ITGA10, ITGA9, ITGB2, ITGB3, JAK1, JAK2, JAK3, JUN, KAT6A, KAT6B, KDM5C, KDM6A, KDR, KEAP1, KIT, KLF6, KMT2A, KMT2C, KMT2D, KRAS, LAMP1, LCK, LIFR, LPHN3, LPP, LRP1B, LTF, LTK, MAF, MAFB, MAGEA1, MAGI1, MALT1, MAML2, MAP2K1, MAP2K2, MAP2K4, MAP3K7, MAPK1, MAPK8, MARK1, MARK4, MBD1, MCL1, MDM2, MDM4, MEN1, MET, MITF, MLH1, MLLT10, MMP2, MN1, MPL, MRE11A, MSH2, MSH6, MTOR, MTR, MTRR, MUC1, MUTYH, MYB, MYC, MYCL1, MYCN, MYD88, MYH11, MYH9, NBN, NCOA1, NCOA2, NCOA4, NF1, NF2, NFE2L2, NFKB1, NFKB2, NIN, NKX2-1, NLRP1, NOTCH1, NOTCH2, NOTCH4, NPM1, NRAS, NSD1, NTRK1, NTRK3, NUMA1, NUP214, NUP98, PAK3, PALB2, PARP1, PAX3, PAX5, PAX7, PAX8, PBRM1, PBX1, PDE4DIP, PDGFB, PDGFRA, PDGFRB, PER1, PGAP3, PHOX2B, PIK3C2B, PIK3CA, PIK3CB, PIK3CD, PIK3CG, PIK3R1, PIK3R2, PIM1, PKHD1, PLAG1, PLCG1, PLEKHG5, PML, PMS1, PMS2, POT1, POU5F1, PPARG, PPP2R1A, PRDM1, PRKAR1A, PRKDC, PSIP1, PTCH1, PTEN, PTGS2, PTPN11, PTPRD, PTPRT, RAD50, RAF1, RALGDS, RARA, RB1, RECQL4, REL, RET, RHOH, RNASEL, RNF2, RNF213, ROS1, RPS6KA2, RRM1, RUNX1, RUNX1T1, SAMD9, SBDS, SDHA, SDHB, SDHC, SDHD, SETD2, SF3B1, SGK1, SH2D1A, SMAD2, SMAD4, SMARCA4, SMARCB1, SMO, SMUG1, SOCS1, SOX11, SOX2, SRC, SSX1, STK11, STK36, SUFU, SYK, SYNE1, TAF1, TAF1L, TAL1, TBX22, TCF12, TCF3, TCF7L1, TCF7L2, TCL1A, TET1, TET2, TFE3, TGFBR2, TGM7, THBS1, TIMP3, TLR4, TLX1, TNFAIP3, TNFRSF14, TNK2, TOP1, TP53, TPR, TRIM24, TRIM33, TRIP11, TRRAP, TSC1, TSC2, TSHR, UBR5, UGT1A1, USP9X, VHL, WAS, WHSC1, WRN, WT1, XPA, XPC, XPO1, XRCC2, ZNF384, ZNF521

Supplementary Table 3: Somatic high-stringency parameters used during next-generation sequencing. See_Supplementary_Table 3

Supplementary Table 4: Protein expression analysed by IHC

Proteins tested	ALK, AR, BAP1, CD8, ENT1, ER, ERBB2, ERBB3, ERCC1, GPNMB, IGF1R, MET, p4EBP1, PARP, PD-L1 (22C3), pERK1/2, PR, pRB, PTEN, RRM1, TERT, TLE3, TOP2A, TOPO1, TS, TUBB3, VEGFR2
