# Supplementary material for: The clinical impact of using complex molecular profiling strategies in routine oncology practice
Source: Oncotarget. 2018 Apr 17;9(29):20282–93. doi: 10.18632/oncotarget.24757 (PMC5945513; doi:10.18632/oncotarget.24757)
Supplement: Supplementary file 2 [file oncotarget-09-20282-s002.docx]

**Supplementary Table 3: Somatic high-stringency parameters used during next-generation sequencing**

| **TSVC Parameters** | | | | |
| --- | --- | --- | --- | --- |
| **Parameter** | **SNP** | **INDEL** | **Description** | |
| min_allele_freq | 0.02 | 0.02 | Minimum allele frequency | |
| min_variant_score | 6 | 6 | Minimum quality | |
| min_coverage | 100 | 100 | Minimum coverage | |
| min_cov_each_strand | 4 | 4 | Minimum coverage on either strand | |
| strand_bias | 0.95 | 0.9 | Maximum strand bias | |
| data_quality_stringency | 10 |  | Minimum relative read quality | |
| filter_unusual_predictions | 0.12 |  | Maximum common signal shift | |
| filter_insertion_predictions |  | 0.2 | Maximum reference/variant signal shift (insertions) | |
| filter_deletion_predictions |  | 0.2 | Maximum reference/variant signal shift (deletions) | |
| **TSVC Advanced parameters** | | | | |
| **Parameter** | | | | **Value** |
| snp_strand_bias_pval | | | | 0.01 |
| position_bias | | | | 0.75 |
| mnp_min_allele_freq | | | | 0.02 |
| mnp_min_variant_score | | | | 6 |
| hp_max_length | | | | 8 |
| heavy_tailed | | | | 3 |
| outlier_probability | | | | 0.01 |
| mnp_strand_bias_pval | | | | 0.01 |
| indel_strand_bias_pval | | | | 1 |
| indel_as_hpindel | | | | 0 |
| mnp_strand_bias | | | | 0.95 |
| position_bias_ref_fraction | | | | 0.05 |
| hotspot_strand_bias_pval | | | | 0.01 |
| sse_prob_threshold | | | | 1 |
| do_mnp_realignment | | | | 0 |
| downsample_to_coverage | | | | 2000 |
| do_snp_realignment | | | | 0 |
| mnp_min_cov_each_strand | | | | 4 |
| mnp_min_coverage | | | | 100 |
| prediction_precision | | | | 1 |
| realignment_threshold | | | | 0 |
| suppress_recalibration | | | | 0 |
| position_bias_pval | | | | 0.05 |
| use_position_bias | | | | 0 |
| min_indel_size | | | | 4 |
| short_suffix_match | | | | 5 |
| min_var_count | | | | 5 |
| min_var_freq | | | | 0.15 |
| output_mnv | | | | 0 |
| max_hp_length | | | | 8 |
| relative_strand_bias | | | | 0.8 |
| kmer_len | | | | 19 |
| gen_min_coverage | | | | 6 |
| allow_mnps | | | | 1 |
| allow_complex | | | | 0 |
| min_mapping_qv | | | | 4 |
| read_snp_limit | | | | 10 |
| allow_indels | | | | 1 |
| read_max_mismatch_fraction | | | | 1 |
| gen_min_alt_allele_freq | | | | 0.01 |
| allow_snps | | | | 1 |
| gen_min_indel_alt_allele_freq | | | | 0.01 |
| **OncoDNA Parameters** | | | | |
| output_filtered | | | | TRUE |
| filter_splice_variant | | | | TRUE |
| filter_synonymous | | | | TRUE |
| min_variant_frequency | | | | 5 |
| minimum_coverage | | | | 100 |
| minimum_variant_coverage | | | | 30 |
| minimum_variant_strand_frequency | | | | 10 |
| minimum_InDel_strand_frequency | | | | 10 |
